# Multilevel Modeling and Policy Development: Guidelines and Applications to Medical Travel

**DOI:** 10.3389/fpsyg.2016.00752

**Published:** 2016-05-24

**Authors:** Eduardo Garcia-Garzon, Peter Zhukovsky, Elisa Haller, Sara Plakolm, David Fink, Dafina Petrova, Vaishali Mahalingam, Igor G. Menezes, Kai Ruggeri

**Affiliations:** ^1^Policy Research Group, Department of Psychology, University of CambridgeCambridge, UK; ^2^Departamento de Psicología Social y Metodología, Facultad de Psicología, Universidad Autónoma de MadridMadrid, Spain; ^3^Department of Psychology, Behavioural and Clinical Neuroscience Institute, University of CambridgeCambridge, UK; ^4^Department of Psychology, Clinical Psychology with Focus on Psychotherapy Research, University of ZurichZurich, Switzerland; ^5^Unit for Paediatric and Adolescent Psychiatry, Division of Paediatrics, University Medical Centre MariborMaribor, Slovenia; ^6^Department of Psychology, Cognitive and Affective Neuroscience, University of ZurichZurich, Switzerland; ^7^Department of Experimental Psychology, Mind, Brain, and Behavior Research Center, University of GranadaGranada, Spain; ^8^Department of Psychology, University of CambridgeCambridge, UK; ^9^Quantitative Methods and Predictive Psychometrics, Institute of Psychology, Federal University of BahiaSalvador, Brazil; ^10^Department of Engineering, Engineering Design Centre, University of CambridgeCambridge, UK

**Keywords:** medical travel, public health, policy, multilevel model, hierarchical linear model, medical tourism, policy research

## Abstract

Medical travel has expanded rapidly in recent years, resulting in new markets and increased access to medical care. Whereas several studies investigated the motives of individuals seeking healthcare abroad, the conventional analytical approach is limited by substantial caveats. Classical techniques as found in the literature cannot provide sufficient insight due to the nested nature of data generated. The application of adequate analytical techniques, specifically multilevel modeling, is scarce to non-existent in the context of medical travel. This study introduces the guidelines for application of multilevel techniques in public health research by presenting an application of multilevel modeling in analyzing the decision-making patterns of potential medical travelers. Benefits and potential limitations are discussed.

## Introduction

In recent years there has been a global increase in international medical travel (Lunt and Carrera, 2010). To date, research on medical travel has been typically focused on patients traveling for treatment unavailable or inaccessible in their home countries (Lunt and Mannion, 2014). Although, country- or region-specific studies are starting to emerge (Johnston et al., 2012; Noree et al., 2014) little is known as to why potential patients travel to specific foreign locations to pursue medical treatment. Given the clear rapid growth of medical travel, understanding which factors influence patients' decisions on a cross-cultural level will provide essential background for the development of effective global health access policies (GHAPs).

A small number of qualitative and quantitative studies have attempted to unravel why individuals choose to travel for medical treatment. However, several shortcomings become evident when reviewing previous work in the psychological and epidemiological literature. First, the extended use of survey methods with a lack of psychometrically validated questionnaires. Second, the primarily descriptive approaches when analyzing medical travel data, which inadequate properties for statistical inference and high-confidence applications. Third, quantitative studies in the field applied traditional statistical methods—as multiple linear regression, that cannot take into account the complex relationship behind the factors analyzed (Gallup Organization, 2007; Varkevisser et al., 2007; Gill and Singh, 2011; Noree et al., 2014). To resolve these issues, better analytic tools combined with access to reliable, validated measurements are needed.

At present, knowledge of factors involved in patient choice is highly dispersed and vaguely connected (Johnston et al., 2012; Hanefeld et al., 2014). Although, available information may offer interesting insights, the findings of most studies cannot be directly integrated, indicating a lack of a robust, comprehensive, and validated model. To unravel the complex decision-making process, psychological drivers and barriers that precede medical travel need to be assessed beyond general economic and availability factors.

Two approaches—revealed and stated preference—have been used to study decision-making processes in economics. Stated preference (SP) is based on the individual's choice between hypothetical scenarios while revealed preference (RP) uses real observations of actual choices in order to infer individuals' preferences. SP studies are based on measurement tools in simulated conditions, where RP are based on observation of individual's choices in real-world setting. Studies suggest (Burge et al., 2005) that stated preference in this context may be used as a reasonable proxy for the measurement of revealed preference.

Each approach has its own strengths and limitations. Revealed preference models may generate results with higher external validity, but are limited to real-world choices, leading to collinearity problems among choices and the need for a high number of observations (Cherchi and Ortúzar, 2007). In contrast, analyzing stated preferences allows researchers not only to create hypothetical alternatives, but also to explicitly examine how different attributes of a given alternative influence the participants' choice, providing a robust and accepted methodology to model choice behavior (Small et al., 2005). Moreover, stated preference models have been widely used in the public health literature and have shown converging results with revealed preference models (Mark and Swait, 2004; Burge et al., 2005; Ryan et al., 2008; Watson and Ryan, 2010).

For the purpose of analyzing stated preference, two main methodologies have been developed: Conjoint Analysis (CA) and Discrete Choice Modeling (DCM). While both frameworks are highly related, in the past decades there has been an increasing support for using DCM over CA as a statistical tool for the analysis of decision-making (Louviere et al., 2010). DCM has also been successfully applied to public health research (Ryan et al., 2012). Detailed explanations of CA and DCM are not provided here and can be found in Louviere et al. (2010) or Small et al. (2005).

The present research design was developed to interpret data such as that from a prototypical DCM experiment in the field, the London Patient Choice Project (LPCP) by Burge et al. (2005). The LPCP investigated the impact of the factors that influenced the decision of choosing between a local and an alternative-hypothetical hospital. The factors included were waiting time, proximity, type of hospital, reputation, and the possibility of follow-up procedures or checks-ups. The design of the study was based on presenting hypothetical scenarios in a DCM design. While LPCP served as an inspiration, several differences can be noted between LPCP and the present study.

The LPCP required participants to choose between two alternative hypothetical scenarios. However, for the purpose of our study, a classical DCM methodology was not directly followed. Instead a hybrid approach was developed by using a psychometric framework focusing on participants' decisions when presented with a hypothetical scenario followed by further inquiry about the motivation behind those decisions.

Moreover, the LPCP and other patient choice studies focused solely on decision-making processes between local hospitals. In contrast, our method encompasses a global approach to the hypothetical scenarios. In particular, by using a country-specific approach (e.g., real locations, rather than hypothetical descriptions), specific country-related evidence can be obtaining, being directly relevant to medical travel policy development. A country-specific approach is obtained by including previously determined countries as attributes in the choice sets. The criteria for country selection can be found in the Medical Travel Data Collection section.

Finally, the LPCP focused on a patient population, similar to most of the literature in the field. The present study is the first of its kind considering a non-patient population. As every individual could be considered as a potential medical traveler, key information regarding which factors drive the decision-making processes could be effectively derived from both populations. While testing prospective choices has several drawbacks, the value of having this knowledge is crucial when developing evidence-based policies related to the implementation of large-scale medical travel programs.

Based on previous findings and the specific research purposes, a new approach to modeling decision-making process in medical travel was developed. This approach examines factors that influence the individuals' decision to travel abroad for non-urgent and essential medical purposes. Its core innovative features include the development of a new medical travel questionnaire based on country-specific hypothetical scenarios (targeting a non-patient population) and the application of a multilevel generalized linear model for analyzing the data collected with the mentioned tool.

The objective of this article is to introduce the new measurement and present the analysis method for medical traveling data by clarifying the theoretical considerations behind the choice of particular multilevel models and presenting results from a case study to highlight its flexibility and usefulness. Furthermore, it is intended to raise awareness of the need for advanced statistical methods when analyzing complex hierarchical structures commonly found in medical travel data (e.g., data from individuals traveling to different countries).

At the same time, the benefits and drawbacks of previous strategies that have been selected for studying decision-making processes underlying medical travel are discussed in comparison to the proposed methodology. Careful use of the data collection method and the statistical analysis could be of benefit not only to GHAP research, but also to other fields that use population measures to support evidence-based policymaking.

## Materials and methods

This study adapted the framework proposed by Louviere et al. (2000) for conjoint experiment design. The present questionnaire relied on a previously validated pathway for the design process and involved the use of four steps as outlined below.

### Step one: defining attributes

#### Specific procedures

Two procedures, hip replacement and heart valve replacement, that were considered as elective, very common, costly or relevant enough to justify traveling were chosen as treatment parameters. Hip and heart valve replacement were considered proxies for invasiveness, with hip replacement being less dangerous or life-threatening than heart valve replacement. Previous studies have shown that patients' decision-making is affected by being in need of a more invasive procedure. In particular, the influence of interpersonal factors is greater when facing such a decision compared to the need for a less invasive procedure (Victoor et al., 2012).

#### Specific countries

The countries included in the questionnaire (see Table 1) are considered potential senders or receivers countries of foreign patients, and play a crucial role in the actual medical travel market. Moreover, they offer a broad range of various healthcare system characteristics. BRICS (Brazil, Russia, India, China, and South Africa) countries have intentionally been excluded due to their unique socioeconomic characteristics, which need to be considered in the future and are currently being studied individually in related work not discussed here.

**Table 1:** **Summary of attributes and their levels**.

**Countries**	**Procedures**	**Driving factors**	**Demographic measures**
Australia	Heart valve replacement	Quality	Previous medical traveling experience
Dubai	Hip replacement	Cost	Home country quality
Germany		Waiting list	Home country cost
Great Britain			Home country waiting list
Malta			Socioeconomic Status
New Zealand			Income
Philippines			Employment status
Portugal			Gender
Qatar			
Singapore			
United States of America			
Thailand			

Traveling abroad for medical reasons requires patients to consider distance and inconveniences caused by physical travel. These factors have been shown to moderate the decision of traveling abroad for medical purposes (Burge et al., 2005; Exworthy and Peckham, 2006). Studies have shown that distance is a key factor, whereby patients (especially elderly patients), prefer closer healthcare providers. Nonetheless, this distance factor is dismissed if a method of easy access is available (by private or public transportation), if the individual is already willing to travel or if that person is highly educated (Victoor et al., 2012). Finally, the effect of distance could be also dismissed or foster depending upon the type of medical procedure under consideration (Damman et al., 2011). Therefore, distance is a highly influential factor on patient's decisions, as indicated in the literature, but is moderated by several second order factors.

#### Specific driving factors

Patients are more likely to travel abroad for medical purposes if the healthcare system in their home country is perceived as low quality, if the healthcare associated costs are considered unacceptable, or waiting times are too long (Burge et al., 2005; Jotikasthira, 2010; Gill and Singh, 2011). Moreover, the quality of the healthcare system has been found to be one of the most prominent indicators in the decision-making process when related to the destination country. Patients also highly value the availability of state-of-the-art technology (Horowitz et al., 2007).

When evaluating alternative destinations, medical travelers reject destinations that they perceive as providing inadequate care quality (Marlowe and Sullivan, 2007). However, people are willing to make a trade-off between the quality of the hospital and potential savings, leading to the choice of a lower quality hospital, if there is potential to save money, under certain circumstances (Jotikasthira, 2010). For instance, medical travelers from industrialized countries including Europe and North America (Gill and Singh, 2011) seek healthcare in less industrialized countries driven by the lower costs (Horowitz et al., 2007). Medical travelers thus consider financial savings as an important factor but only when there is also an acceptable quality standard of medical care (Jotikasthira, 2010).

Shorter waiting lists are also an important factor for individuals when considering going abroad for medical purposes, but only after considering the healthcare system's quality (Burge et al., 2005). Previous research provides evidence relative to how patients' motivation to travel for medical care is boosted if waiting time for medical travel can be reduced (Ryan and Gerard, 2003; Burge et al., 2005). The importance given to this potential reduction of the waiting list however depends on the medical condition of the patient (Ricketts et al., 2004; Exworthy and Peckham, 2006) and on the proximity of the new provider (Burge et al., 2005). Nevertheless, waiting list has also been consistently listed as a relevant factor in the existing literature (Burge et al., 2005; Jotikasthira, 2010; Johnston et al., 2012).

Individuals are generally most willing to travel for access to higher-quality treatment, followed by potential savings and the avoidance of long waiting lists (Burge et al., 2005; Gallup Organization, 2007; Keckley and Underwood, 2008; Jotikasthira, 2010). As argued by Exworthy and Peckham (2006), this may be due to a search for convenience, and thus understanding of the interaction of these variables along with trade-offs is very important. Important trade-offs are made between these specific factors and the home country's healthcare features. As a result, satisfaction with the perceived quality, cost, and waiting time of the home country's healthcare are assessed in order to obtain information relative to these trades-offs.

#### Demographic questions

In addition to the promise of better quality, reduced costs and shorter waiting lists certain demographic characteristics are also known to play an important role in the decision to travel for medical care.

Employment status, income, and occupation are considered to be proxy measurements of socioeconomic status (SES) (American Psychological Association, Task Force on Socioeconomic Status, 2007). Highly educated patients and patients with higher income (Burge et al., 2005; Exworthy and Peckham, 2006) make an active choice of health care provider compared to patients without those characteristics (Victoor et al., 2012). Individuals with higher educational level (Exworthy and Peckham, 2006; Lako and Rosenau, 2009) are also more likely to travel abroad for medical purposes.

Previous medical travel experiences (Varkevisser et al., 2007; Victoor et al., 2012) are also an important moderator of patients' decisions, where individuals tend to rely on their previous experience as the most important information source (Kolstad and Chernew, 2009; Lux et al., 2011).

### Step two: creating scenarios

The chosen attributes were combinations of one of two procedures, one of three driving factors, and one of 12 destination countries. An example scenario would be:

“*Imagine your doctor says you require heart valve replacement, yet the waiting list is long in your home country. However, in Malta, the waiting list is considerably shorter. Under these circumstances, would you consider traveling to Malta for this procedure?”*

Attempting to simulate the real medical decision-making process as much as possible, the logic behind the scenario' structure is:

1. Patient receives the information about his medical condition and the necessity of a medical procedure.
2. Patient seeks information related to the conditions of the medical procedure in his home country.
3. Patient considers alternative countries that provide a better situation for that medical procedure.

Presenting the participant with a real-world based situation creates the possibility to capture generalizable trade-offs between factors, simulating realistic patient's decision making processes.

### Step three: determining scenario sets

CA/DCM studies use factorial or fractional factorial designs, as they need to present two scenarios with a different set of characteristics at the same time for analyzing the differential factors behind the decision making processes. This results in an unmanageable number of possible combinations between scenarios and attributes (Louviere et al., 2000). In contrast, the proposed medical travel questionnaire is based on the presentation of only one scenario at a time, allowing a complete randomization of the possible 72 scenarios across participants, generating balanced conditions for each attribute.

The medical travel questionnaire was designed as an on-line questionnaire, prepared to be adapted to the contingencies associated by the data collection in social media. Qualtrics software was used for the generation of blocks randomly assigned to participants, once the country of origin was taken into consideration. Participants did not receive items assessing their home country, as home country was not fully randomized between participants. A country was therefore randomly assigned—only controlling for home country to be excluded—and remained constant across the combination of procedure and reasons, so that participants answered more than one item for a given country.

Block randomization involved developing blocks of six questions to ensure that all participants encounter all possible combinations between factors and procedures within a randomly given country. For each driving factor (quality, cost, and waiting list), two questions regarding the two procedures (hip or heart valve replacement) were generated. All the questions in each given scenario are explicitly referred to one country.

The decision to include only six scenarios can be justified by several means. Firstly, measurement fatigue leading to withdrawal was considered a legitimate concern if participants were presented with the all possible (72) scenarios. Furthermore, it is not necessary for patterns to emerge if the large sample is sufficiently large, which would make for even more insightful results if participants are unlikely to recognize that each scenario is entirely unique and not simply a collection of all possible permutations.

Therefore, the trade-off between a large inventory of responses per participant and a large sample of participants answering fewer items is a significant decision but may be modified in future versions if resources or samples are available to complete the entire questionnaire. Given the nature of the data and current purpose of this approach, it is considered preferable to have a large number of participants answering a smaller number of items.

### Step four: estimating model parameters

#### Why should multilevel models be applied in medical travel research?

Traditional statistical methods such as multiple linear regression have dominated quantitative research in medical travel. They provide valuable information regarding the different factors involved in medical travel, but these are limited to local settings and often hold unrealistic assumptions about the data analyzed. Medical travel is an international phenomenon that can be structured in different layers and levels of action. Fittingly, multilevel models are the proper analytical tool for taking into consideration these classes of structures, providing information regarding all the units involved. No traditional technique would therefore be adequate when analyzing medical travel data.

Classical statistical tools assume that all variables are constrained in one level of analysis, where all the predictors are hypothesized to be characteristics within the same level of analysis (Snijders and Bosker, 2012). However, this is not the case when studying the influence of the country of destination in the decision-making processes for medical travel. Due to advances in modeling approaches, the assumption of independence between observations is no longer a valid axiom in statistical analysis, as demonstrated by the increasing number of studies that use multilevel methods for analyzing the interdependencies among observations.

Here, multilevel methods are defined as the set of techniques that allow the estimation of parameters in different levels of the data in order to take into account the nested structure of the observations in a given dataset. The flexibility of the multilevel method is achieved by defining hierarchical structures where higher-level units capture the dependency among units in the lower level. Therefore, instead of assuming independency between observations, the researcher can explore those relationships by the inclusion of higher-level units, enhancing the quality of the information given by the model by providing an explicit explanation of first level units' shared variance.

Although, several names can be found in the literature for referring to these models (such as hierarchical linear models a random-effects models) here we will refer to them as multilevel methods (MLM). One of the main theoretical advantages of MLM is its ability to capture the country-specific effects arising from differences in regional characteristics that modulate the individual's response in a decision-making processes. At the same time, MLM relies on less strict assumptions than conventional DCM models and does not require non-independence among observations, hence providing a solution to the multicollinearity problem that is so common to questionnaire-based datasets. Snijders and Bosker (2012) recommended the use of the intraclass correlation coefficient (ICC) for examining the amount of variance explained by second level units in a model, where values ranging from 0.05 to 0.2 are common in cross-sectional social science research (Peugh, 2010).

Failing to take into account these interdependencies leads to underestimation of the standard deviation of the regresssion coefficients and dramatic increase of Type 1 error (Austin et al., 2003). As the ICC grows, standard deviation and parameter estimations tend to be inaccurate (Julian, 2001), generating potentially incorrect predictions and misleading conclusions. In addition, it is mandatory to take into consideration the higher-level units (e.g., countries) when disclosing medical travel patterns and processes. Conclusions drawn from classical techniques are based on biased estimation of the defined parameters (and incorrect standard error estimations), potentially leading researchers to draw misinformed conclusions and misguided recommendations for practitioners. Multilevel methods provide the solution for adjusting parameter estimation to the hierarchical nature of the data by modeling the existent interdependencies.

As country-specific scenarios were generated, a hierarchical model where country of destination was the second-level variable could be easily derived. Multilevel logistic models allow the study of the non-independent relationships between destination country and a set of fixed effects while placing the focus on these country-related random effects. Furthermore, country-specific information can be obtained when using these methods, simultaneously clarifying the complicated interactions between factors in the decision-making processes. This information is not only richer, but also more accurate than that obtained by using traditional methods, allowing the estimation of fixed and random effects along with their standard errors (Snijders and Bosker, 2012).

An exhaustive review of multilevel methods is beyond the scope of this article, and important features of multilevel logistic methods are omitted here. Interested readers may refer to the following literature (Pinheiro and Bates, 2000; Hox, 2002; Raudenbush and Bryk, 2002; Gelman and Hill, 2007).

Previous empirical evidence generated by multilevel models in medical travel has not been found using DCM/CA paradigms, but multilevel logistic methods have been widely used in other areas of public health research (Rice and Leyland, 1996; Duncan et al., 1998). Moreover, the effect of country as a second-level unit has been specifically analyzed (Stegmueller, 2013) and robust evidence exists to justify its use in this analytical method. The multilevel approach presented here is the first application of multilevel methodology to medical travel data.

#### Mathematical definition of the multilevel model

The model under consideration is a two-level random intercept model. The individual's response *i* (*i* = 1,…, *nj*) for a country *j* (*j* = 1, …, *J*) is modeled (γ_ij_)as follows:

$$\gamma_{ij}\;=\;ln\frac{pi_{ij}}{1\;-\;pi_{ij}}\;=\;\beta_{0}\;+\;\beta_{1}x_{1j}\;+\;\beta_{2}\;x_{2j}\;+\;\ldots \;+\;\beta_{k}x_{kj}\;+\;u_{0j}\;+\;e_{ij}$$

$$u_{0j}\sim N(0,\Omega_{u}):\Omega_{u}=[\sigma_{j}^{2}]$$

$$e_{ij}\sim N(0,\Omega_{e}):\Omega_{e}=[\sigma_{e}^{2}]$$

$$cov\left(u_{0j},e_{ij}\right)=0$$

The individual's decision to travel is assumed to be binomially distributed, where the logit of the probability of traveling (π_*ij*_) abroad depends on a linear combination of the model intercept (β_0_), a set of k individual-level predictor variables x_ij_—with their β_i_ coefficients—the random error term at individual level (e_ij_) plus the random error term at context level (*u*_0*j*_). Both error terms are assumed to be normally, identically $u_{0j}\sim N\left(0,\Omega_{u}\right):\Omega_{u}=\left[\sigma_{j}^{2}\right];\;e_{ij}\sim \;N\;\left(0,\Omega_{e}\right):\Omega_{e}=\left[\sigma_{e}^{2}\right];$ and independently distributed (*cov* (*u*_0*j*_, *e*_*ij*_) = 0). The distributions are defined as diagonal matrices with variance components in the diagonal elements and with zeros in the off-diagonal elements.

Individual-level variance is fixed as $\sigma_{e}^{2}$ = $\frac{\pi^{2}}{3}$, as recommended by Goldstein et al. (2002) or Rasbash et al. (2009). The model is expressed in terms of the mean or expected value of γ_*ij*_ for an individual *i* in a group *j* with a value *x*_*ij*_. Individual terms predictors include the studied procedure, driving factors (e.g., how the individual decision changes when considering two different procedures) and sociodemographic features described in the previous section, plus relevant interactions among the variables at this level (e.g., if the influence of the considered procedure varies when considering potential savings, the quality or the waiting time for that specific operation).

A two-level random intercept model is sensitive to differences across country-level units, while concurrently holding all the effects constant across countries (Gelman and Hill, 2007), as random effects are only modeled regarding the error terms. Therefore, the effect of the included individual level variables is modeled as fixed for each given country. This model provides valuable information while representing the simplest model that could be derived. In order to generate evidence of the existence of country-specific trends in medical traveling previous to extent this methodology. Further developments could include random slopes models and more complex variance models (Rasbash et al., 2009), or alternatively revert to universal scenarios for parallels and predictors of pragmatic designs.

Although the random-intercept model is considered to be the most parsimonious of its class, it provides valuable information regarding the usefulness of the hierarchical linear model given the context. Firstly, a random-intercept model is the basis for demonstrating the necessity of multilevel models in a given data prior to the application of more complex multilevel models. Secondly, information about both, fixed and random effects could be obtained with an unbiased parameter estimation. The parameter estimates (and the corresponding estimation error) contain the relevant information for explaining the different trade-offs between the reasons, procedures and countries of destination, and how each of them are related to the decision of going abroad for medical care. Consequently, this model is expected to offer richer information than classical techniques for both, fixed and random effects defined therein. Multilevel logistic models allow not only parameter estimation for fixed and random effects, but also for cross-level effects, which is an important improvement over traditional multivariate analysis. The specific effect of driving factors (e.g., the impact of the cost of the procedure on the decision making process) or even the effects of second level units characteristics (e.g., geographical location of the country) can be estimated along with interactions between countries' and the driving factors or procedures They are not included in the previous formulation of the model for the sake of simplicity, but could be easily be incorporated to the model if needed, following the references previously provided.

## Case study: application to medical travel data

### Introduction to Zhukovsky et al. (2015) medical travel data

Following the aforementioned approach, the proposed framework for collecting and analyzing medical travel was applied. An illustration of the methodology is provided in order to ease the comprehension of the previous theoretical approach, and to encourage researchers to confront the results thereupon presented with the evidence provided by alternative methods. Hence, both data and results presented hereafter are intended to be considered only as an example of the possibilities and flexibility of the presented method.

The method was illustrated by using data presented in Zhukovsky et al. (2015), and readers interested in further details on the dataset are referred to contact the authors of the paper. The data was collected under the auspice of the Global Health Access Project, developed by the Junior Researcher Programme, in May and August 2014. An online questionnaire was developed following the specifications abovementioned on this article. This dataset consisted of 3174 observations obtained from 529 participants, where the main characteristics of the sample can be found in Table 2. Only complete observations were included in the model, following the analysis decisions originally made by the authors in Zhukovsky et al. (2015). The decision to travel abroad (individual's response to undergo for medical care to a given country) differed across various attribute categories (Table 3), where procedure, reason and countries of destination were identified as the most influential factors both from a theoretical and statistical point of view. As indicated by Zhukovsky et al. (2015), an interaction between procedure and reason was observed and was included in the multilevel model presented here.

**Table 2:** **Characteristics of study sample**.

**Variable**	**Value**	**Freq. %**	**Mean (SD)**
**GENDER**			
	Female	67.8	
Age			27.6 (10.36)
**MEDICAL TRAVEL EXPERIENCE**			
	Yes	5.0	
	No	95.0	
**REGION OF ORIGIN**			
	Europe^*^	87.7	
	Other	12.3	
**EDUCATION**			
	Master Degree, PhD or eq.	43.6	
	Bachelor Degree or eq.	30.9	
	Secondary School	19.0	
	Higher Vocational Training	6.5	
**INCOME**			
	Very high	1.7	
	Above average	22.1	
	Average	43.5	
	Low	29.8	
	Below poverty	2.9	
**TIME ABROAD**			
	No time abroad	62.1	
	Between 6 months and 1 year	14.5	
	More than a year	23.3	
RATING OF LOCAL HEALTHCARE^**^			
	Quality		3.7 (0.98)
	Waiting		2.7 (1.13)
	Cost^**^		3.5 (1.11)

* Europe = Germany, Slovenia, UK, Spain, and other countries

** *Values refer to a Likert scale ranging from one to five, with 1 = very negative, 2 = negative 3 = neutral, 4 = positive, 5 = very positive*.

**Table 3:** **Frequency of agree to travel abroad due to medical purposes**.

**Condition**	**Value**	**Percentage of agreement to travel (%)**
**OVERALL**		
	Would agree to travel	66.9
**BY PROCEDURE**		
	Hip Replacement	64.0
	Heart Valve	69.8
**BY REASON**		
	Quality	82.0
	Waiting Time	63.5
	Cost	55.4
**BY COUNTRY**		
	Australia	72.8
	Dubai	66.0
	Germany	91.6
	Great Britain	89.1
	Malta	70.0
	New Zealand	73.7
	Philippines	41.1
	Portugal	68.0
	Qatar	57.8
	Singapore	56.7
	United States of America	74.0
	Thailand	42.5

Descriptive results (Table 3) show that individuals generally agree to travel for medical care, but the conditions surrounding that decision could play an important role for the final decision. Each individual responded to six questions that consisted of all six possible combinations of three procedures and two reasons and each of the six questions featured randomly assigned countries of destination. This design invites a hierarchical model where responses are nested within country. Therefore, the demographic variables, procedure, and reason are included in the model as first level variables (e.g., they are defined as fixed effects), nested in the country of destination (second level procedures, defined as random terms). Moreover, in order to illustrate the abovementioned approach, the consequent analysis will be restricted to be a random-intercept model with country of destination as the unique random term.

### Random intercept model

Four multilevel models were included in the results. First, the null or unconditional model was created, including only the intercept and the random term (country of destination). Second, the unconditional model was expanded by including the main interest effects (procedure and reason). Thirdly, the interaction between the main effects was included, as it has been previously shown to play an important role in this analysis. Lastly, demographic variables described in Table 2 were included in the model as first level covariates to serve as control variables for the analysis of the main effects. Moreover, an additional single-level model was included for comparison purposes. This model includes all the fixed effects from the final model without the random term.

The reference categories were identified as follows: for procedure, the less invasive procedure (i.e., hip replacement) was selected as the reference category. For reason, cost was the reference category, as it was the most balanced procedure in terms of agreement and disagreement with traveling abroad for medical purposes. Female gender was the reference category for the named variable. Income and education were centered on their mode category (Income = Average income; Education = Master's degree, PhD, or equivalent). Three variables related to the evaluation of the home country's healthcare system were centered on their mean values. Any other variable was fixed to the categories that represented the absence of the referred variable (e.g., for “Previous Medical Travel Experience” the reference category was “No”).

All the models were estimated using restricted maximum likelihood with Gaussian-Hermite quadrature integration (10 integration points). ICC measures were provided in order to evaluate the percentage of variance due to the random effect. In order to evaluate the appropriateness of the specified models, AIC, BIC, and likelihood tests are used. R software (R Development Core Team, 2015) and lme4 package (Bates et al., 2015) were chosen as the software platform for performing the analysis.

### Case study results

Parameter estimations for the fixed and random effects, as well as ICC, AIC, BIC, and likelihood ratio are presented in Table 4.

**Table 4:** **Fixed effect estimates (top) and random effect estimates (bottom) for models of the predictors of agreeing to travel**.

**Parameters^a^**		**Null model**	**Main effects**	**Main effects and interaction**	**Final model**	**Single-level model**
**FIXED EFFECTS**						
**Level 1**						
Intercept		2.24 (1.26)^**^	1.12 (1.27)	1.33 (1.29)	4.71 (1.62)	5.73 (1.49)^**^
**Procedure**						
	Heart valve replacement		1.36 (1.08)^**^	0.97 (1.14)	1.00 (1.15)	0.98 (1.14)
**Reason**						
	Waiting List		1.46 (1.09)^**^	1.01 (1.14)	0.99 (1.15)	1.00 (1.14)
	Quality		4.26 (1.1)^**^	3.76 (1.16)^**^	3.93 (1.15)^**^	3.52 (1.16)^**^
**Procedure** × **Reason**						
	Heart valve replacement × Waiting list			2.19 (1.21)^**^	2.13 (1.22)^**^	2.17 (1.21)^**^
	Heart valve replacement × Quality			1.27 (1.24)	1.31 (1.25)	1.27 (1.23)
**Income**						
	Below Poverty				0.64 (1.36)	0.49 (1.34)^*^
	Low				0.84 (1.18)	0.75 (1.17)
	Above Average				0.67 (1.20)^*^	0.80 (1.19)
	Very High				0.30 (1.48)^**^	0.37 (1.45)^**^
**Time Abroad**						
	Between 6 months and 1 year				3.57 (1.19)^**^	3.17 (1.17) ^**^
	More than a year				7.18 (1.25)^**^	6.64 (1.23) ^**^
**Rating of local healthcare**						
	Waiting list				0.75 (1.10)^*^	0.75 (1.09)^**^
	Cost				1.30 (1.09)^*^	1.19 (1.08)^*^
	Quality				64 (1.10)^**^	0.6 (1.10)^**^
**Education**						
	Secondary School				1.73 (1.21) ^**^	1.4 (1.20)
	Vocational School				1.25 (1.22)	1.05 (1.21)
	Bachelor Degree or eq.				0.44 (1.21) ^**^	0.45 (1.19) ^**^
**Previous medical travel**						
	Experience in medical travel				0.61 (1.28)^*^	0.59 (1.26)^*^
**Gender**						
	Male				0.82 (1.17)	0.8 (1.16)
**RANDOM EFFECTS**						
**Level 2 (Country of destination)**						
Intercept		1.80 (2.16)	1.93 (2.24)	1.94 (2.24)	1.99 (2.29)	–
**Model summary**						
ICC		–	16.70%	16.77%	21.07%	–
Deviance statistic		3774	3488.1^**^	3470^**^	3294.8^**^	3590.1^**^
AIC		3708	3498.1	3527.1	3336.8	3630.1
BIC		3720.8	3528.4	3484.8	3463.9	3751.2

a All the results are presented as odd ratios with standard errors in parentheses

* *Significant at 0.05 level*.

** *Significant at 0.01 level*.

Since adding interactive effects of procedure and reason and demographic effects improved the model fit, only the final model was interpreted. Firstly, ICC results shows that almost 20% variance in the first level units is accounted by the effect of the country of destination, demonstrating the necessity of taking this hierarchical structure into consideration for performing unbiased parameter estimation. Similarly, both AIC and BIC were decreasing within the nested models, and likelihood-ratio test is highly significant when comparing this model with a model without the fixed term demographic effects. Therefore, all the indicators reflect the adequacy of both the random intercept model and the model including the fixed term covariates for our data.

Results show that decisions to go abroad for medical purposes were influenced by several factors, and that those relationships were complex, with several factors interacting with each other.

Firstly, the odds of going abroad if a higher quality of the treatment was expected was 3.93 times the odds of agreeing to medical travel when considering the effect of the procedure's cost. Quality seems to be the most compelling factor when deciding to go abroad for medical traveling or not.

Moreover, an interactive effect between the procedure and the reason was found. When the procedure under consideration was heart valve replacement, the odds ratio between going abroad for this procedure was 2.13 times greater when considering shorter waiting time than when pondering costs. This effect was not found to be significant when the odds ratio were referring to the difference between the odds of deciding to go abroad for medical care between heart valve and hip replacement for quality and cost. Hence, it was observed that when individuals are faced with a highly invasive procedure (e.g., heart valve replacement), the associated time pressure is a highly relevant factor. However, when the procedure is less risky and less life threatening, the monetary cost become as important as waiting time. The interactive effect of waiting time or cost conditional upon the procedure was less important the main effect of quality, which is highlighted as the most influential of all the reasons analyzed here.

Demographic variables provide important information for creating profiles of medical travelers. Firstly, gender was not an influential variable in the decision-making process. Secondly, the odds of going abroad for medical care for individuals with high incomes are lower than the odds for those with average income by a 70%. Thirdly, the more educated an individual is, the less likely they are to decide to opt to receive medical treatment abroad. The odds of travel abroad for medical purposes for people who have only completed secondary school were 1.73 times higher than the odds for individuals with a Master or PhD or equivalent. Following the inverse relationship found to exist between education and medical traveling, the odds of agreeing to travel for care for individuals who completed their bachelor degree or equivalent were a 56% lower than the odds for individuals with a Master's degree, PhD, or equivalent.

Another important predictor of how likely an individual would be to decide to opt for medical travel is whether that individual has previously lived abroad. The odds of traveling for people living abroad for a time between 6 months and 1 year are 3.57 times higher than the odds of going abroad for medical purposes in individuals who spent less than 6 months abroad. Furthermore, the odds of deciding for medical travel for someone who has lived abroad more than 1 year are 6.18 times the odds of deciding for medical travel for someone who has not lived at least 6 months outside his/her home country.

Individual's perception of their own country's healthcare system was expected to be another important factor underlying their decision to undergo a medical procedure abroad. Again, the rating of the quality of the quality of the healthcare system is the most influential factor, as the odds for going abroad for medical care are decreased by 36% for each one-point increase of the quality rating. Furthermore, the odds of deciding for medical travel decreased by 25% with each increased point when evaluating waiting times. Surprisingly, the opposite effect was found when participants were asked to evaluate the cost of the healthcare system in their home country, whereby the odds of traveling increased by 30% for each increased point. While the effects of the rating of the cost and waiting time of the home healthcare are significant, they are less important (by means of the estimated odd ratio comparison) than the evaluation of the healthcare system's quality, which played a greater role than the other aspects of the healthcare in participants' home countries.

Lastly, previous medical travel experience was found to be negatively related with the decision to travel abroad for care, as the odds of an individual with previous experience are only 0.61 times the odds of deciding to travel for individuals without any previous medical travel experiences.

Regarding the comparison with the single-level model, BIC results indicated that the final multilevel model fits the data better than the single-level model. Given the known problems of both the likelihood-ratio test and AIC (Richards, 2005; Bolker, 2008), it is advised to proceed with caution when interpreting them. Both techniques have been shown to be extremely conservative when comparing linear mixed effects models, but no information regarding their behavior in generalized linear mixed models was found. In order to provide as many insightful results as possible, likelihood-ratio test and AIC were presented. Estimates for fixed effects for the single-level model were expected to be similar to those found in the final multilevel model condition that could occur when comparing models with highly balanced structures. In this case, the randomness of the given country when providing the test to the participants generated a structure where the number of observations per country was similar (*M* = 2.61, min 249, max 268). Estimated standard errors were smaller for the single-level model than for the final multilevel model. The differences between the standard errors of both models is due to the failure for the single-model to take into account the non-independence of the observations. The inclusion of the random term in the final multilevel model lead to less biased estimation (Bolker, 2008). Therefore, both empirical and theoretical considerations support the use of the final multilevel model.

### Case study discussion

The multilevel approach previously presented is not only important for providing a correct estimation of the fixed parameters, but also for providing estimates of random effects of country of destination (Table 5). These results allow us to map trends on the medical travel market, whereby participants are more likely to agree to travel to north European countries (Germany and United Kingdom) and the USA for medical care. Australia and New Zealand complete the map of the most compelling countries when deciding to seek medical attention abroad. Southern Europe (Portugal and Malta) are less preferred destination for receiving medical care. On the other hand, Middle East (Dubai and Qatar) are not deemed as compelling options. Lastly, individuals tend to choose not to travel to countries in Southern-Asia (Thailand, Philippines and Singapore) for medical purposes.

**Table 5:** **Percentage, estimated odd ratios, and estimated probability of participants willing to travel for care for each country**.

**Country**	**Sample agreement to travel (%)**	**Estimated odds ratios for travel**	**Estimated probability of travel (%)**
Australia	72.8	1.28	56.1
Dubai	66.0	0.86	46.4
Germany	91.6	4.19	80.7
Great Britain	89.1	3.65	78.5
Malta	70.0	1.01	50.3
New Zealand	73.7	1.38	58.0
Philippines	41.1	0.29	22.3
Portugal	68.0	1.00	50.0
Qatar	57.8	0.62	38.3
Singapore	56.7	0.55	35.7
Thailand	42.5	0.28	21.6
United States of America	74.0	1.49	59.8

This case study outlines some of the advantages of the multilevel method. Firstly, multilevel modeling can generate unbiased parameter estimates for the fixed effects when a hierarchical structure are present in the data. In addition to random effect estimation, multilevel models can also measure the variance that is explained by those random terms by means of the ICC, becoming an indicator of the importance of those random effects. Lastly, multilevel modeling critically improves upon the classical statistical techniques by increasing the inferential power of the analysis and by taking into account the effects of higher-level variables that arise due to the nested structure of the data. A direct comparison between the multilevel model mentioned above and a single-level model illustrates how the inclusion of random effect terms can improve the model fit and lead to less biased estimates.

Direct comparison with other tools (i.e., the LPC) is difficult and controversial, as the DCM estimation techniques differ radically from those employed in multilevel methods. Several challenges arise when attempting such a direct comparison. Firstly, neither background theories supporting our study or DCM studies (that usually encompass utility theory), nor the data collection methods are relatable, hence allowing for only speculative conclusions from these comparisons. Ignoring such caveats would lead to the situation of comparing results from statistical methods commonly derived from the generalized linear family and regression analysis, in a situation where those results could not be directly comparable without a common theoretical framework (that to our notice, not psychologist, not econometricians have developed yet). Nevertheless, as both classes of studies are based on statistical methods from the generalized linear family, the aforementioned benefits of using multilevel regression methods apply to both types of studies, and the presented recommendations could be also extended to those studies. Even without a direct comparison of these methods, it is possible to point out the advantages of multilevel models over DCM methods and their usefulness to various types of medical travel data.

Lastly, several limitations could also be identified in the dataset analyzed here. Above we only presented the case for the random-intercept model. It includes the assumption that every first level effect is fixed to be constant across all the second level units, but this assumption might not hold in real-world datasets. Therefore, researchers should be aware that complex models (e.g., random-slopes, random-intercept and random-slopes, multilevel with cross-levels effects) could represent alternatives that are more realistic. Sadly, the inclusion of new random effects comes with the price that more second level units should be incorporated in the model and that larger samples are needed.

In addition, 90% of the participants were from European countries, which reduces the generalizability of our findings to populations outside Europe regardless of the statistical technique used. Further discussion of this dataset and its limitations can be found in Zhukovsky et al. (2015).

## Discussion

### Implication for medical travel data analysis

The motivation behind this article was to demonstrate a new method not only for the study of the decision-making processes in medical travel, but also the exploration of existing trade-offs involved in that decision, aiming to improve the quality of the research methods in the field relevant to health policy.

A new questionnaire has been theoretically implemented along with a complementary analytical method. Both are the result of the application of widely used techniques in other research areas (i.e., CA/DCM studies) to psychology and public health. This approach aimed to transfer these methodologies to our field of interest to improve the quality of the generated evidence. We argue that multilevel analyses provide richer inferences due to its greater power, hence generate more insightful evidence that can be used by policymakers to adjust their interventions. In addition, the multilevel approach helps elucidate the complex relationships between the studied moderators and a certain set of countries of destination. The effect of a destination country on the effects of the other variables on the decision-making process can now be individually analyzed. This goes significantly beyond what can be inferred from theoretical, artificial destinations or care options for direct use in application.

As illustrated by the case study, the application of multilevel modeling can provide reliable estimation of the coefficients for various variables, providing trustworthy information about how the different procedures and reasons interact to enhance or discourage individual's decision of going abroad due to medical reasons. Moreover, the effect of demographic factors (i.e., gender, education, or income) allows the development of medical traveler profiles.

Multilevel methods are increasingly common in social science data analysis due to their flexibility for assessing relationships among different units of analysis and their moderators, but have so far been rather scarce in policy research (Cascio and Aguinis, 2008). In the future, more widespread application of these more complicated, yet more sensitive models could serve to increase the reliability and power of public health policy research studies. In addition, identifying country-specific trends and patterns could help international agencies provide most relevant information to patients and national governments.

As researchers, our ultimate objective is to provide trustworthy information to governmental agencies, stakeholders and patients, leading to the generation of evidence-based policies. New methodologies should be implemented for and by researchers to be able to analyze complex relationships and moderated effects in both psychology and public health areas. Medical traveling is becoming a global and significant phenomenon, with important economic and social consequences. Its growing importance is reflected in the increasing number of publications in this area. Investing time and effort in developing new research designs would only revert on social gains, as the quality of the implemented policies grow. Consequently, the personal benefits of the implementation of these policies for clients and users would lastly be fostered.

Lastly, the research presented was intended to illuminate the benefits and the caveats of the application of multilevel modeling to medical travel phenomena. Four main conclusions may be drawn from this study:

1. There is a soundly theoretical ground for justifying the implementation of multilevel modeling in medical traveling. When studying social phenomena involving hierarchical structures with several units (i.e., different procedures, different countries, different traveling patterns), research should seek for applying the appropriate statistical methods (i.e., multilevel modeled).
2. The use of multilevel modeling does not only provide more accurate information of each of the second-level units (e.g., each of the countries), but is also able to disclose the complex relationships between the unit of the model and the predictors of the model. Therefore, specific and unique information can be obtained by using multilevel modeling (e.g., variance explained by the random effects), which is not accessible in an accurate fashion by means of using traditional techniques.
3. This information can be directly extrapolated to national agencies and shareholders, and could have a direct impact on the decision-making processes and policy development. Multilevel models do not only provide a more realistic representation of the medical travel, but encompasses the analysis of medical travel data into a well-known statistical framework.
4. When applied to empirical data, the use of multilevel model allowed an unbiased estimation of the random and the fixed effects and their standard deviations, being superior to its single-level counterpart (see Appendix A in Supplementary Material). Results confirmed that individuals are more likely to travel to countries in Northern Europe (England and Germany) and less likely to go to Southern-Asia countries. Regarding which factors drive the decision-making processes in medical traveling, treatment's quality was the most compelling factor. When individuals faced non-invasive procedures (i.e., hip replacement), the treatments' costs were considered as prominent as the waiting lists. Contrarily, when individuals confronted life-threating procedures (i.e., heart valve replacement), the waiting time was seen as more influential factor than the treatments' cost. In addition, individuals with lower SES, who had lived abroad for more than 1 year, with no previous medical traveling experience and with negative views of the quality, cost and waiting times from their own healthcare system were more likely to travel abroad for medical care.

### Further developments

The development of strong theoretical approaches is a priority for gathering scientific knowledge, but it needs to be confirmed with real data in medical travel. This study introduced a new data collection method, highlighting its strengths and limitations and criticized the lack of appropriate statistical techniques in the field of health policy. Instead, we propose and defend multilevel modeling as a promising way of detecting effects that so far have been overlooked due to the inadequacy of traditional DCM methods. The finding that estimated probabilities of agreeing to travel to a given country differ from descriptive evidence provides sound evidence of the importance of the fixed effects in the data. Multilevel methods are an excellent method of exploring these effects since they offer greater power to detect small and possibly interactive effects that may be driven by higher-level units such as destination country, at the same time relaxing the assumption of non-independence.

In addition, the sample of participants and/or countries involved should also be representative of the population under study and specific to the research question. Studies targeting different populations and different travel trends could be carried out under the proposed method with small modifications. Nevertheless, 90% of the analyzed sample was formed by individuals from European countries. Having such a high percentage of the sample located in a “single” world region could be stated as a limitation in both the original and this case study.

The proposed method is just an approximation to the rich world of multilevel analysis. Further developments should include a generalization of this method using other complex multilevel architectures. Moreover, random slope, or a random intercept and random slope model—considering the inclusion of cross-levels interactions—should be analyzed in future research. These new models could be implemented to generate new evidence in the field of medical travel. Particularly interesting would be the development of models with cross-levels effects including procedures, reasons, countries of destination and home country of the participants, in order to properly identify trends in medical travel, mapping the current medical markets and their evolution. Presenting an example of the previously theoretically developed approach, it is expected that researchers on the field would feel encouraged to explore not only the aforementioned alternatives, but to incorporate the study of multilevel models in their own research.

## Author contributions

KR designed and supervised the project as well as all writing. EG defined the scope and objectives of the paper, plus was the primary contributor of writing. EG, PZ, EH, SP, DF, and KR reviewed the literature on the field and designed the data collection. EG, PZ, IM, DF, and VM provided the technical expertise. EG, KR, SP, EH, and DP wrote the paper. All the authors (EG, PZ, EH, SP, DP, DF, IM, VM, and KR) reviewed the manuscript, approving the final version of the paper.

### Conflict of interest statement

The authors declare that the research was conducted in the absence of any commercial or financial relationships that could be construed as a potential conflict of interest.
