# Supplementary material for: Multilevel Modeling and Policy Development: Guidelines and Applications to Medical Travel
Source: Front Psychol. 2016 May 24;7:752. doi: 10.3389/fpsyg.2016.00752 (PMC4877536; doi:10.3389/fpsyg.2016.00752)
Supplement: Supplementary file 1 [file DataSheet1.docx]

**Appendix A.**

**Guidelines for multilevel model applications**

In this section, readers will find the following information (a) the theoretical distinction between mixed effects and hierarchical models,(b) the number of 2^nd^ level units required for estimating a multilevel model, (c) the selection of the proper estimation method, (d) choosing between likelihood ratios vs Wald test, (e) statistical software for estimating multilevel methods.

First, all hierarchical methods are special cases of the multilevel model family, as all the multilevel models are definitions of broader mixed-effects models. Necessarily, all hierarchical multilevel logistic models are mixed effects models (as they include random effects), though not all mixed effects models are hierarchically structured (Gelman, 2006; Pinheiro and Bates, 2000), leading to several confusions in the literature between hierarchical, multilevel, and random-effects methods. In the present context, we explicitly contrast a hierarchical model in which the responses are clustered by the country of destination, with a possible logistic mixed effects regression. Although the use of the aforementioned data collection method usually results in a nested structure of the data, statistical indicators of hierarchical structure in the data provide a good validation of the statistical method choice.

Second, when considering a random intercept hierarchical linear model, a minimum sample size of 15-20 countries (i.e. second level units) should be aimed for in order to avoid bias in maximum-likelihood estimates and confidence intervals for second level units. A large sample of individual-level units could ensure correct individual-level estimates (Stegmueller, 2013). More complex variance structures would require larger numbers of second level units at both levels. In addition, the use of Bayesian techniques could lead to an accurate estimation of fixed effects with a lower number of second-level units, when compared with maximum-likelihood techniques (Hox, van de Schoot&Matthijsse, 2012).

Third, when considering different parameter estimation methods, maximum likelihood could be preferred over restricted maximum likelihood method as this technique allows comparison across models with different fixed effects (Bolker, 2008; Peugh, 2010). Restricted maximum likelihood has been shown to be more accurate when dealing with small number of second-level units (Wolfinger and O’Connell, 1993). Within maximum likelihood methods, Gaussian-Hermite quadrature seems to be the most accurate option when working with small number of random effects (Pinheiro and Chao, 2006). Laplace Approximation as well as Gaussian-Hermite quadrature is recommended over Penalised-Quasi likelihood (Bolker, 2008). Moreover, estimation could be improved via parametric and semi-parametric bootstrapping. Bayesian estimation (via Monte Carlo Markov Chains) offers considerable advantages over their analogous frequentist competitors, as optimal estimation under small sample size conditions (Austin et al., 2010).

Fourth, when considering model selection, likelihood ratio tests are an improvement over Wald test (Singer and Willett, 2003) while deviance criterion is even more preferable when working with Monte Carlo Markov Chains for parameter estimation, as it penalizes model complexity.

Last, almost all statistical packages facilitate the integration of these methods in their software. Following Zhang et al., (2011), SAS packages NLMIXED and GLIMMIX or R packages nlmeand lme4 are recommended, but statistical software as MLwiN or HLM also are attractive alternatives. Researchers should be aware of the limitations and capabilities of each statistical software, as not all of them provide the necessary tools for estimating models with complex estimation techniques.

**Further considerations regarding the case study results.**

The previous guidelines when using multilevel models are generalizable to any multilevel model. While well-documented and extensive discussions regarding different aspects of multilevel models already exist, we present a comprehensive set of further clarifications regarding their application to medical travel in the Zhukovsky et al., (2015) data. These clarifications include details on: (1) selecting the appropriate data structure, (2) details on model fit and how to compare a multilevel model with its single-level counterpart, (3) diagnostic and analysis plots and (4) interpretation of the model.

First, further exploration of how different hierarchical structures would further influence the effect’s estimations could be appropriate. In an alternative analysis of the data, the structure of the Zhukovsky et al., (2015) data could potentially be specified as cross-classified model, or a multiple membership model (i.e. each scenario crossed in countries and individuals). Researchers should be aware of the implications of selecting different types of structure, supporting their decisions on both technical and theoretical aspects. Some reasons to consider when choosing between two different structures include data limitations (e.g. with a small number of second level units crossed-classified models estimates could be biased), the research question at hand, and the data collection plan. Further developments of this work are expected to expand and compare the presented model with alternative specifications of the structure, once a sufficient number of second level units is achieved.

Second, information regarding the multilevel model fit and appropriateness should be always be verified. In order to understand if a multilevel model is indeed supported by the data, AIC, BIC and ICC provides statistical justification of its use. AIC and BIC provide evidence regarding better statistical fit of the multilevel model when compared with an alternative, single level model. ICC indicates how much variance is due to the inclusion of the second level units. Results showed that the inclusion of the countries of destination as a random effect was strongly justified. First, 20.07% of the variance of the model was explained by the second level units. Second, both AIC and BIC were lower for the random-intercept model than for the single-level model.

Third, diagnostic analysis and plots should be always consulted, in order to understand if the logistic multilevel model is the appropriated model for this data. Following Bolker et al., (2009), it useful to check if the link function represents the appropriate error distribution, and to obtain evidence of possible overdispersion and the use of alternative models (such as zero-inflated models) for dealing with those contingencies. Pinheiro and Bates (2000) also provides useful resources for model evaluation, and suggest the use of visuals tools such as qqplots, location-scale plots and to graph the random and fixed effects by using caterpillar graphics.

Fourth, after careful inspection of the model’s fit and adequacy to the data, researchers should face the interpretation of the model. Practitioners should know where to obtain the precise information they are seeking for. If the focus of their research is to obtain information regarding the importance of the factors included in the model (i.e. as occurs in the presented case), random and fixed effects should be examined. When analyzing the discrepancies or similarities of the fixed effects estimates obtained from the multilevel and the single-level model, it is clear that fixed effects estimates have been corrected in the random-intercept model, especially for those fixed effects with unbalanced responses (similar to the differences in estimation when analyzing data by no or complete pooling; Gelman, 2006). As an example, the difference in the estimation for the highly balanced parameter (i.e “Procedure”) was minimal ($\Delta= .02).$ For this effect, the number of answers to scenarios containing heart valve replacement (50.06% of the sample) or hip replacement was similar. Moreover, the ratio of affirmative responses to medical travel was similar for heart valve replacement (2.30 affirmative responses for each negatives) and for hip replacement (i.e. the reference category for this effect; 1.89).

If researchers intend to better understand how the model predicted the probability of traveling for each individual (i.e. with the intention of classifying subjects or to create profiles of medical travelers), the predicted responses should be computed. These predicted responses could be compared with the original data. In the case under study, differences were rather small (largest difference = 1.3%), and shrinked the estimated probabilities towards the overall mean.
